# Genome-wide identification and expression analysis of the PHD-finger gene family in *Solanum tuberosum*

**DOI:** 10.1371/journal.pone.0226964

**Published:** 2019-12-27

**Authors:** Mingyue Qin, Wenbin Luo, Yan Zheng, Huazhong Guan, Xiaofang Xie

**Affiliations:** 1 College of Life Sciences, Fujian Agriculture & Forestry University, Fuzhou, China; 2 Key Laboratory for Genetics, Breeding and Multiple Utilization of Crops, Ministry of Education, Fujian Agriculture & Forestry University, Fuzhou, China; 3 Fujian Key Laboratory of Crop Breeding by Design, Fujian Agriculture & Forestry University, Fuzhou, China; 4 The Crop Institute, Fujian Academy of Agricultural Sciences, Fuzhou, Fujian, China; Laboratoire de Biologie du Développement de Villefranche-sur-Mer, FRANCE

## Abstract

Plant homeodomain (PHD) proteins are prevalent in eukaryotes and play important roles in plant growth, development and abiotic stress response. In this study, the comprehensive study of the PHD family (*StPHD*) was performed in potato (*Solanum tuberosum* L.). Seventy-two *PHD* genes (named *StPHD1-72*) were identified and grouped into 10 subfamilies based on phylogenetic analysis. Similar structure organizations were found within each subfamily according to the exon/intron structures and protein motif analysis. These genes were unequally scattered on the chromosomes of potato, with 9 pairs of segmental duplicated genes and 6 pairs of tandem duplicated genes showing that both segmental duplicated and tandem duplicated events contributed to the expansion of the potato PHD family. The gene ontology (GO) analysis suggests that *StPHD* mainly functioned at the intracellular level and was involved in various binding, metabolic and regulation processes. The analysis of expression patterns of *StPHD* genes showed that these genes were differentially expressed in 10 different tissues and responded specifically to heat, salt and drought stress based on the FPKM (Fragments per kilobase of transcript per million mapped reads) values of the RNA-seq data. Furthermore, the real-time quantitative PCR for 12 selected *StPHD* genes revealed the various levels of gene expression corresponding to abiotic stress. Our results provide useful information for a better understanding of *PHD* genes and provide the foundation for additional functional exploration of the potato *PHD* gene family.

## Introduction

Zinc-finger proteins are widely dispersed in eukaryotic organisms. Zinc-finger domains are rich in cysteine or histidine and have been classified into several types, including RING (Really Interesting New Genes), LIM (Lin11, Isl-1 and Mec-3), and plant homeodomain (PHD) [1]. The majority of PHD proteins are in the nucleus [2]. A typical PHD protein usually contains one to several PHD-finger domains, and each of the proteins contains approximately 60 amino acids with the structure composition of Cys4-His-Cys3 zinc-binding motif [3]. The residues of the pair of cysteines or between cysteine and histidine are usually conserved, whereas the second amino acid residue before the final pair of cysteines is usually tryptophan or another aromatic amino acid [4]. The combination of the core amino acid residues and two zinc ions plays a fundamental role in maintaining a firm spatial framework for the domain, a roughly globular domain in a three-dimensional conformation [5]. PHDs proteins have been widely studied in plant species since the first PHD protein HAT3.1 was identified in *Arabidopsis* [6]. Studies have shown that PHD proteins perform critical roles in plant development [7]. In Arabidopsis, the PHD protein VIL1 regulates the expression of floral repressors through photoperiod and vernalization pathways. DUET, a PHD protein, is also essential for chromosome organization and progression during spermatogenesis in Arabidopsis [8]. The PHD protein VIM1 contains the histone residue and participates in the regulation of the chromatin state [9]. As a PHD domain containing protein, *SIZ1* codes a SUMO E3 ligase, which contains the PHD domain and regulates the anther dehiscence for spikelet fertility. Furthermore, *SIZ1* regulates both vegetative and reproductive development in plants [10, 11]. The *Ehd3* gene, which encodes a PHD protein, is an important promoter of rice flowering [12]. Recent studies showed that PHD proteins are essential epigenetic members in methylation maintenance [13, 14]. For example, the PHD proteins ATX1 and ATX2 control the expression of genes that encode histone methyltransferase [15]. In addition, some PHD proteins are involved in abiotic stress response [16]. Six soybean PHD proteins, encoding the Alfin1-type PHD protein, showed differential expression in response to cold, ABA and drought treatment, and displayed resistance to salt stress in transgenic Arabidopsis [7].

The *PHD* gene families have been studied in several plant species, such as Arabidopsis (*Arabidopsis thaliana*), maize (*Zea mays*) [17], poplar (*Populus trichocarpa*) [18], Chinese pear (*Pyrus bretschneideri*) [19] and bamboo (*Phyllostachys edulis*) [20]. Potato (*Solanum tuberosum*) is one of the most important food crops in the world, with more than 380 million tons of field production worldwide (http://www.fao.org). However, the *PHD* gene family in potato has not been thoroughly examined. Genome sequence and annotation resources of the potato genome provides an excellent opportunity for understanding the *PHD* gene family [21]. In this study, a comprehensive investigation of the *PHD* gene family in the potato genome was conducted, including *PHD* gene structure, chromosomal localization, gene duplication, phylogenetic relationship, gene ontology, tissue-specific expression profile, and expression patterns following exposure to drought, heat and salt stress. The objectives of this study were to identify the genome-wide sequence structures and the evolutionary relationship of the potato *PHD* gene family and provide a better understanding of functional mechanisms of the PHD-finger gene family in plants.

## Materials and methods

### Identification of *PHD* genes

The PHD protein sequences of *Arabidopsis thaliana* were used as the queries to identify the amino acid orthologs in potato through the BLASTP tool of SpudDB [22] and Phytozome v12.1 (https://phytozome.jgi.doe.gov). Potato candidate PHD proteins were identified with more than 30% similarity to the query sequence and an E value less than E^-10^. The genomic sequences of *Arabidopsis thaliana* PHD proteins were downloaded from the Phytozome database (https://phytozome.jgi.doe.gov/pz/portal.html). The domains for PHD proteins were confirmed by using the Conserved Domain Database (CDD) from NCBI (https://www.ncbi.nlm.nih.gov/cdd/) [23] with an E value of 1e^-10^. Finally, the sequences with complete PHD domains were retained and were assigned as potato PHD (*StPHD*) genes. The information for gene IDs, physical position, sequence of gene and protein, and coding sequences (CDS) were retrieved from Phytozome. These *StPHD* genes were renamed according to the order of their physical position.

### Gene structure and conserved motif identification

The parameters for the final confirmed PHD proteins were calculated using online ExPASy programs (http://web.expasy.org/protparam/) [24]. The exon-intron structures for the *StPHD* genes were identified on the Gene Structure Display Server (GSDS, http://gsds.cbi.pku.edu.cn/) [25]. The conserved motifs were identified by the MEME program (version 5.0.3, http://alternate.meme-suite.org/tools/meme) [26] using an optimum motif width of 50–250 residues and 20 as the number of maximum motifs. The function of the identified motifs was annotated with the CDD program [23].

### Chromosomal location and gene duplication

The chromosomal location image of *StPHD* genes was generated by the MapInspect tool (http://www.plantbreeding.wur.nl/uk/software_mapinspect.html) based on gene physical position data. Gene duplication was determined by the length and similarity (>70%) [27, 28]. Tandem duplicated genes were defined when the physical interval of two duplicated gene was less than 100 kb and they were separated by less than five genes [29].

### Phylogenetic analysis

Alignment of all potato PHD-finger protein sequences was generated using ClustalX1.83 [30]. A phylogenetic tree with 1,000 bootstrap replicates was constructed based on the IQ-TREE maximum-likelihood method [31]. BLASTN was used to identify the orthologs among potato, Arabidopsis and maize [32]. The two sequences with the best alignment were confirmed as orthologs if the alignment was more than 300 bp and shared at least 40% similarity.

### GO annotation and RNA-seq data analysis

GO analysis for *StPHD* genes was performed using PlantRegMap (http://plantregmap.cbi.pku.edu.cn/go.php). FPKM values of *StPHD* for various tissues and treatments derived from RNA-seq (DM_v4.03) [21] were extracted from SpudDB. The heatmap2 function of the R package for heatmap functions were used to analyze the expression of *StPHD* genes [33].

### Plant growth and treatments

T Virus-free plantlets (*S*. *tuberosum* L. autotetraploid cultivar Zhongshu 3) were produced from in vitro cuttings. Using the nodal cutting method, the potato shoots were cultured in full MS solid media with a 16-h day temperature of 22°C and an 8-h night temperature of 18 °C for one month. The plantlets were then grown in a tray with a half-strength modified Hoagland solution [34] for 6 days prior to treatment. Small foam squares were used to suspend plantlets in the solution, which was adjusted to cover the roots. Plantlets were exposed to abiotic stress conditions included heat (35°C), drought (260 mM mannitol) and salt (150 mM NaCl) treatment, untreated plantlets were used as control groups. All treated plantlets and controls were collected 6 h after treatment and stored at −80°C before RNA samples were extracted.

### Quantitative real-time PCR analysis

The total RNA of the plantlets was extracted using TRIzol reagent (Invitrogen, http://www.invitrogen.com). The cDNA samples were assayed by qRT-PCR using SYBR Premix Ex Taq (Takara). The primers used for qPCR analysis are listed in S1 Table. *Actin* was used as an internal control. Three biological replicates (each replicate contained 6 plants) and three technical replicates were tested. The relative expression level of a gene was measured using the 2^−ΔΔCt^ method [35].

## Results

### Identification and characterization of *PHD* genes

A BLASTP search was performed using 70 PHD protein sequences of Arabidopsis [36] as the queries against the potato genome database to analyze *PHD* genes in potato. In addition, the CDD analysis was used to validate the presence of the PHD conserved domain in the predicted sequences. Finally, a total of 72 *PHD* genes were confirmed in potato and these genes were named *StPHD1* through *StPHD72* based on their physical position on chromosomes (Table 1). However, a single gene (*StPHD1*) was located on the unassembled scaffolds, and therefore, *StPHD1* could not be assigned to any of the potato chromosomes. The *StPHD* genes varied greatly in terms of the number of amino acids and molecular weights. The StPHD proteins contain 115 (*StPHD42*) to 1,569 (*StPHD38*) amino acids, with an average of 532.7 amino acids, whereas the molecular weights ranged from 12.94 kDa (*StPHD42*) to 175.35 kDa (*StPHD38*) and the theoretical isoelectric points (pI) varied between 4.43 (*StPHD24*) and 9.27 (*StPHD63*). The detailed information for *StPHD* genes, including the gene ID, protein length (aa), molecular mass (MS), pI and location are listed in Table 1.

**Table 1:** The *PHD* family genes in *Solanum tuberosum*.

Gene name	Gene ID	Protein			Chr.	Location
		Length (a.a.)	Mol. wt. (Da)	PI		
*StPHD1*	PGSC0003DMG400006740	173	18752.74	6.18	0	39070649‥39071170
*StPHD2*	PGSC0003DMG400022773	531	60482.71	9.01	1	381797‥384537
*StPHD3*	PGSC0003DMG400022774	531	60328.35	8.72	1	389334‥392000
*StPHD4*	PGSC0003DMG400006859	1167	129839.47	6.09	1	65467581‥65472212
*StPHD5*	PGSC0003DMG400024731	949	108238.64	4.92	1	77859513‥77863125
*StPHD6*	PGSC0003DMG400018261	248	28022.56	5.16	1	79431944‥79437244
*StPHD7*	PGSC0003DMG400025760	578	63075.32	4.78	1	83891354‥83895400
*StPHD8*	PGSC0003DMG400030955	211	24452.44	8.49	1	86394125‥86398488
*StPHD9*	PGSC0003DMG400018039	707	78191.68	7.13	1	88623560‥88626720
*StPHD10*	PGSC0003DMG400021118	545	60035.47	7.01	2	29095938‥29104284
*StPHD11*	PGSC0003DMG400016712	211	24070.4	8.46	2	36806739‥36811222
*StPHD12*	PGSC0003DMG401012812	748	84165.62	8.67	3	1914594‥1918304
*StPHD13*	PGSC0003DMG400022537	822	88752.29	5.20	3	3774837‥3781249
*StPHD14*	PGSC0003DMG400016917	919	104398.78	8.05	3	16816173‥16820858
*StPHD15*	PGSC0003DMG400011962	709	77804.29	5.43	3	22309009‥22311760
*StPHD16*	PGSC0003DMG400009121	704	80208.21	8.87	3	45237890‥45246090
*StPHD17*	PGSC0003DMG400018162	643	73457.51	8.86	3	52582102‥52584033
*StPHD18*	PGSC0003DMG400000605	735	84248.62	6.14	3	56433707‥56436222
*StPHD19*	PGSC0003DMG400002485	276	31569.31	4.73	3	61241418‥61245409
*StPHD20*	PGSC0003DMG400006006	705	78358.88	8.39	4	1614152‥1617748
*StPHD21*	PGSC0003DMG400029502	534	60406.38	5.78	4	3056100‥3058915
*StPHD22*	PGSC0003DMG400029488	747	83866.01	5.24	4	3252237‥3254776
*StPHD23*	PGSC0003DMG400037898	514	58420.76	7.81	4	3281893‥3284133
*StPHD24*	PGSC0003DMG402018008	153	17300.49	4.43	4	56927171‥56927632
*StPHD25*	PGSC0003DMG400018006	289	32109.28	4.83	4	56954320‥56955189
*StPHD26*	PGSC0003DMG400009357	595	67593.61	6.28	4	65066151‥65070283
*StPHD27*	PGSC0003DMG400033921	686	76008.65	7.17	5	17769678‥17773971
*StPHD28*	PGSC0003DMG400027429	678	77234.89	6.36	5	19370632‥19373940
*StPHD29*	PGSC0003DMG401017043	228	26080.79	8.45	5	32754127‥32757896
*StPHD30*	PGSC0003DMG400008456	549	61964.65	6.74	5	49835834‥49840825
*StPHD31*	PGSC0003DMG400016262	153	16795.42	5.45	6	40408827‥40409456
*StPHD32*	PGSC0003DMG400016768	320	36468.83	9.03	6	45161128‥45168547
*StPHD33*	PGSC0003DMG400020472	362	41318.5	8.65	6	45612751‥45616624
*StPHD34*	PGSC0003DMG400026134	319	36736.1	7.53	6	45906361‥45911506
*StPHD35*	PGSC0003DMG400037001	280	31695.41	6.63	6	45930651‥45935015
*StPHD36*	PGSC0003DMG400004887	257	29094.65	5.05	6	48587673‥48593031
*StPHD37*	PGSC0003DMG400004060	650	71454.44	8.79	6	51123204‥51129858
*StPHD38*	PGSC0003DMG400007126	1569	175353.5	7.48	6	55175748‥55182922
*StPHD39*	PGSC0003DMG400028740	719	81505.82	8.52	7	6333109‥6341436
*StPHD40*	PGSC0003DMG400020951	247	28051.45	4.99	7	37507602‥37511402
*StPHD41*	PGSC0003DMG400002391	347	39664.27	8.09	7	40086321‥40087538
*StPHD42*	PGSC0003DMG400009849	115	12938.17	8.37	7	40131259‥40131606
*StPHD43*	PGSC0003DMG400006146	546	61748.02	5.81	7	44316143‥44320770
*StPHD44*	PGSC0003DMG400000673	234	26735.49	6.50	7	44939178‥44941263
*StPHD45*	PGSC0003DMG400031096	386	43398.3	7.88	7	51461135‥51463722
*StPHD46*	PGSC0003DMG400007086	302	33717.54	6.02	7	52252668‥52254002
*StPHD47*	PGSC0003DMG400012431	485	53833.92	5.19	7	52735489‥52742279
*StPHD48*	PGSC0003DMG402022202	823	89381.03	8.39	7	55159113‥55164799
*StPHD49*	PGSC0003DMG400026258	622	71489.42	8.60	8	51235299‥51240722
*StPHD50*	PGSC0003DMG402012227	946	102296.14	7.90	8	56760683‥56768601
*StPHD51*	PGSC0003DMG400003902	942	103697.19	8.77	9	4612598‥4621802
*StPHD52*	PGSC0003DMG400023078	241	27297.64	5.32	9	8326536‥8334789
*StPHD53*	PGSC0003DMG400022079	1217	138563.93	6.54	9	39798638‥39807441
*StPHD54*	PGSC0003DMG400023100	354	39514.2	7.74	9	42979927‥42982358
*StPHD55*	PGSC0003DMG400030174	406	45444.27	9.19	9	43275771‥43285203
*StPHD56*	PGSC0003DMG400030175	194	21554.59	8.00	9	43358605‥43359881
*StPHD57*	PGSC0003DMG400041970	235	26104.01	5.56	9	43720347‥43721437
*StPHD58*	PGSC0003DMG400032262	466	51807.81	6.25	9	53864208‥53868363
*StPHD59*	PGSC0003DMG400011588	236	26231.55	6.14	9	57716591‥57724943
*StPHD60*	PGSC0003DMG400019178	240	27090.43	5.77	10	50195563‥50201617
*StPHD61*	PGSC0003DMG400011022	240	27074.37	5.18	10	53903104‥53908590
*StPHD62*	PGSC0003DMG400011059	514	56806.2	7.35	10	54485212‥54491878
*StPHD63*	PGSC0003DMG400028181	767	84820.19	9.27	10	55326036‥55335829
*StPHD64*	PGSC0003DMG400023718	869	95197.69	5.59	10	57644472‥57648188
*StPHD65*	PGSC0003DMG400008367	258	29818.38	4.96	10	59176774‥59180143
*StPHD66*	PGSC0003DMG400013249	167	18644.64	8.05	11	1051899‥1052552
*StPHD67*	PGSC0003DMG400036423	333	37045.91	4.52	11	1088487‥1089488
*StPHD68*	PGSC0003DMG400013247	683	77481.46	5.66	11	1102178‥1104593
*StPHD69*	PGSC0003DMG400028656	395	45262.077	8.12	11	6978554‥6982962
*StPHD70*	PGSC0003DMG400027792	445	49761.58	8.12	11	9596045‥9597382
*StPHD71*	PGSC0003DMG400000459	1059	116446.3	4.95	11	39273198‥39281957
*StPHD72*	PGSC0003DMG400037817	1328	150283.02	6.52	11	41080332‥41083612

### Phylogenetics and structure of PHD protein in potato

**Fig 1:**
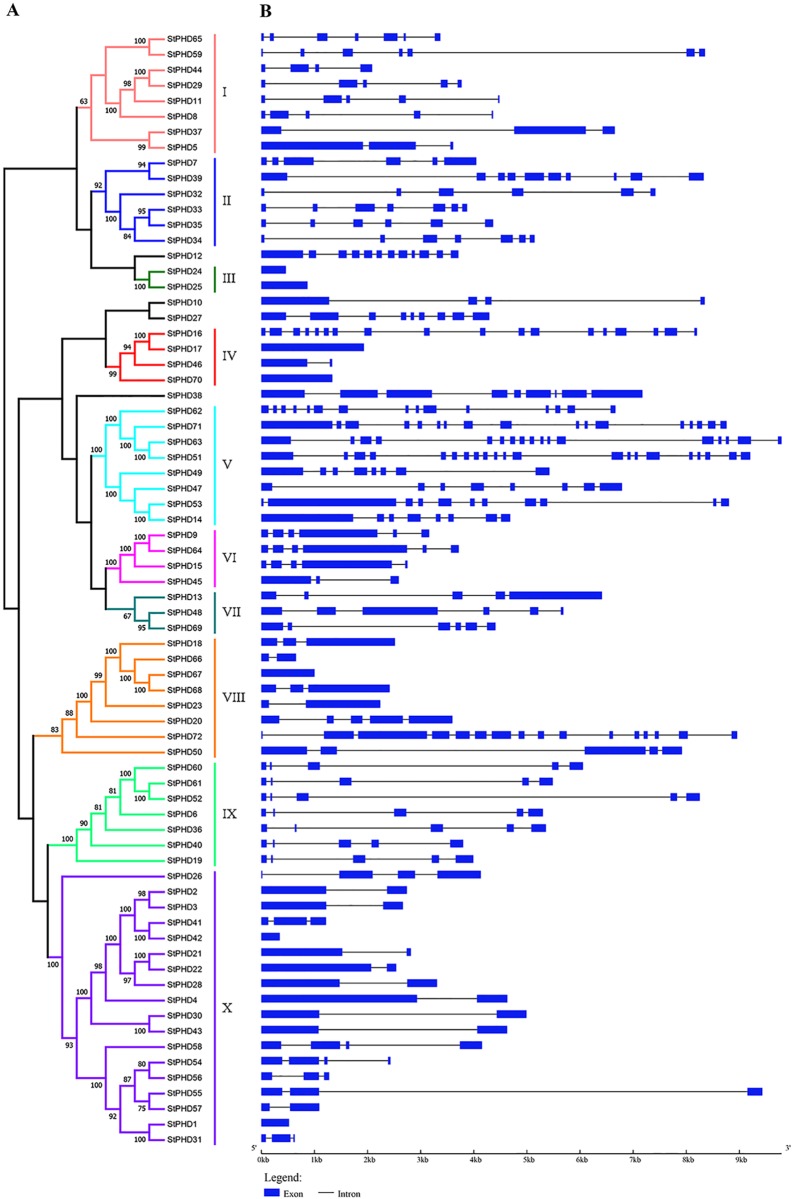
Phylogenetic relationships and gene structures for the StPHD family. A. Phylogenetic tree generated by maximum-likelihood method with bootstrapping analysis (1,000 replicates) based on the protein sequences of *StPHD* genes (percentage of bootstrap value was displayed at each node). The branches of different groups are shown in different colors. B. The graphic displays exon-intron structures for *StPHD* gene family members using GSDS. The horizontal black lines and the blue boxes show introns and exons, and the scale displays the relative length and position of the introns and exons.

Phylogenetic analysis was performed, and an unrooted phylogenetic tree was generated using the 72 potato PHD protein sequences, with 1,000 bootstrap replicates, to reveal the evolutionary relationship for the PHD family (Fig 1A). Moreover, the exon/intron structures for individual *PHD* genes were investigated (Fig 1B). Together with the results of gene structure analysis, the *StPHD* family genes were clustered into 10 groups (I through X) with the bootstrap values (≥60%) on the phylogenetic tree. However, 4 *StPHD* genes (*StPHD12*, *-10*, *-27* and *-38*) could not be grouped into any subfamily for the 10 groups due to the low bootstrap values (<60%). Among these 10 groups: group X was the largest group, which contained 18 members; group I, V and VIII had 8 members; group IX had 7 members, and these five clades represented 68.06% of the total StPHD proteins. In contrast, groups III and VII only contained two to three members.

Structure analysis of *StPHD* genes (Fig 1B) showed the number of introns ranged from 0 to 20. Among them, 7 genes had no introns (*StPHD24*, *-25*, *-17*, *-70*, *-67*, *-42* and *-1*) and *StPHD51* had the largest number of introns (20). Most of the *StPHD* genes that shared the same group showed similar exon/intron structure, including the intron numbers and exon length, and however, exceptions were also found among these genes. For example, *StPHD* genes in group I contained 2 to 6 introns, and the members in *StPHD* gene groups II contained 5 to 9 introns. Interestingly, the members in group V had larger gene sequences and contained a wide range of introns (7–20) whereas they exhibited great diversity in exon length.

### Potato PHD-finger domain and motifs

To further investigate the similarity between the potato PHD-finger domains, 72 PHD-finger domain sequences were aligned (S1 Fig). The highlighted figures in black and pink colors were the Zn ion binding sites with seven cysteines and one histidine residue (Cys4-His-Cys3), and the result indicated that the PHD-finger domain of potato is very conservative. Based on the result of alignment, the consensus sequence of the potato PHD-finger domain was C–X(1–2)-C–X(4–24)-C–X(2–17)-C–X(4–15)-H-X2-C–X(2–25)-C–X(0–4)-C. The consensus sequence of potato PHD domain was basically consistent with previous research [17, 18, 37]

**Fig 2:**
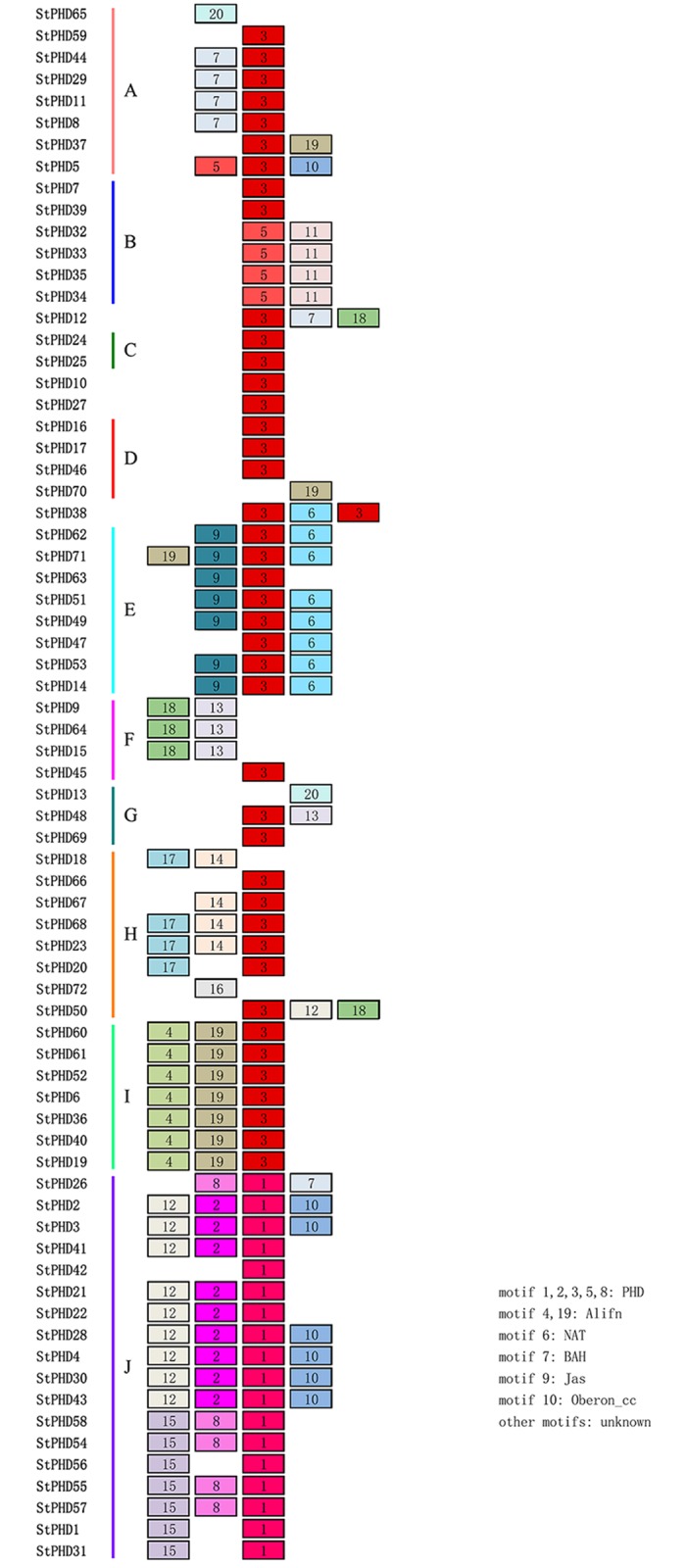
Schematic diagram of conserved motifs for StPHD proteins. Different motifs are displayed with different colored boxes and numbers (1–20). The annotation of each motif listed on the right, which was identified by CDD program.

The online MEME software was used to investigate the conserved motifs of 72 StPHD proteins within each subfamily to investigate the diversity of motif components among *StPHD*s. A total of 20 distinct motifs were identified (Fig 2), and the detailed sequences for each motif is listed in S2 Table. By searching CDD database, 11 putative motifs were acquired functional annotation and motifs 1, 2, 3, 5 and 8 were annotated for the components of the conserved PHD domain (Fig 2), and however, no functional annotation was found for the other 9 putative motifs. The majority of StPHD proteins in the same subfamily have similar motif components and distribution, suggesting that the StPHD proteins share the same groups may possess similar functions. For example, proteins in group I possessed motifs 4, 19 and 3, while group E members contained motifs 9, 3 and 6. However, significant differences were also observed between different groups, and some motifs were exclusively presented in a particular group, suggested that these motifs might play specific functions in the group. For instance, motifs 4 and 19 contain a histone-binding component, which is common for all members in group I and play an important role in transcription start [38]. Motifs 6 and 9 present in group E members, and motif 6 is a type of N-Acetyltransferases (NAT) and mostly catalyzes the transfer of an acyl group. Motif 9 possesses Jas domain and this motif appears to bind to the Groucho/Tup1-type co-repressor TOPLESS (TPL) and TPL-related proteins (TPRs) and with an important role in the jasmonate signalling pathway [39]. Motif 7 is the Bromo-adjacent Homology domain (BAH) in group A members and plays an important role in DNA methylation, replication and transcriptional regulation [40]. While many members in group J contain motif 10, it is Oberon Coiled-coil region (Oberon_cc) and plays an important role for maintenance and establishment of both the shoot and root apical meristems [41].

### Chromosomal locations and duplications of *StPHD* genes

**Fig 3:**
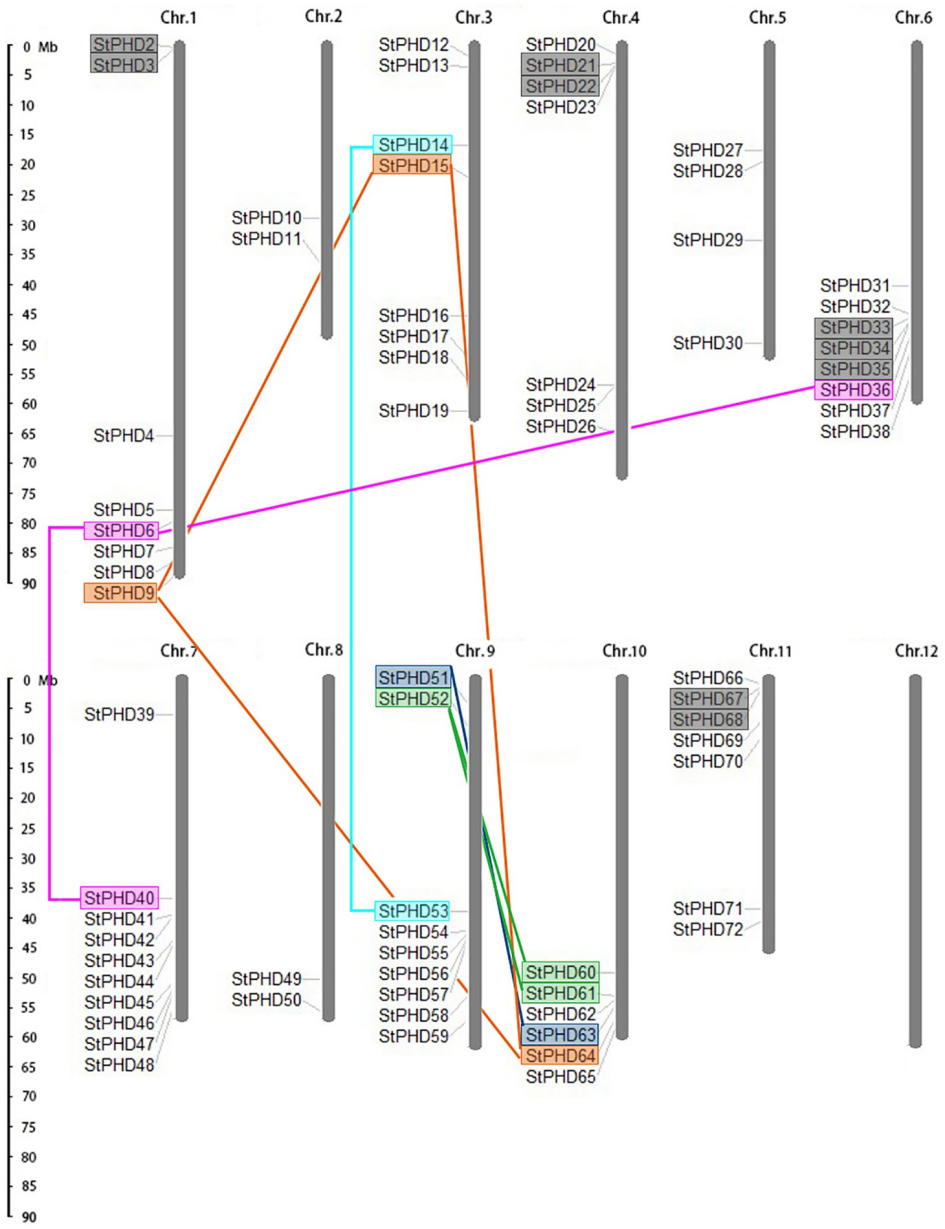
Chromosomal locations of potato *PHD* genes. The left scale represents the length of potato chromosomes (Mb). The tandem duplicated gene pairs are marked with gray boxes, and the segmental duplicated gene pairs are marked with colored boxes and are linked by corresponding color lines.

A total of 71 out of the 72 *PHD* genes were assigned to the 11 potato chromosomes, except chromosome 12 (Fig 3). Chromosome 7 contained the largest number of *PHD* genes (10), followed by chromosomes 9 (9 *PHD* genes), whereas chromosomes 2 and 8 possessed the smallest number of *PHD* gene members (2). A similar uneven distribution pattern of *PHD* gene families was also observed in Arabidopsis [36] and *Populus trichocarpa* [18]. The regions on the proximate or the distal ends of potato chromosomes exhibited a dense distribution of *StPHD* genes, such as the bottom of chromosomes 1, 6, 7, 9, 10 and 11. Both tandem duplication and segmental duplication perform an important role in the expansion of the gene family [42]. The potential duplication events were investigated to reveal the detailed mechanism for the *PHD* gene family. According to the phylogenetic and comparative analysis of the *StPHD* genes, 9 gene pairs (*StPHD6/40*, *StPHD 9/15*, *StPHD 6/36*, *StPHD 9/64*, *StPHD 15/64*, *StPHD 14/53*, *StPHD52/60*, *StPHD52/61* and *StPHD51/63*) were found to be involved in the segmental duplication events (Fig 3). A biased distribution pattern was also found among the 9 segmental duplication pairs and no pairs were distributed on chromosomes 2, 4, 5, 8, 11 and 12. In addition, 6 gene pairs (*StPHD2/3*, *StPHD21/22*, *StPHD67/68*, *StPHD33/34*, *StPHD33/35* and *StPHD34/35*) were confirmed to be tandem duplicated genes, and they were distributed on chromosomes 1, 4, 5 and 11. These results suggest that segmental duplication events and segmental duplication may play important roles in the amplification of the potato *PHD* gene family.

### Phylogenetic analysis of the *StPHD* gene family

**Fig 4:**
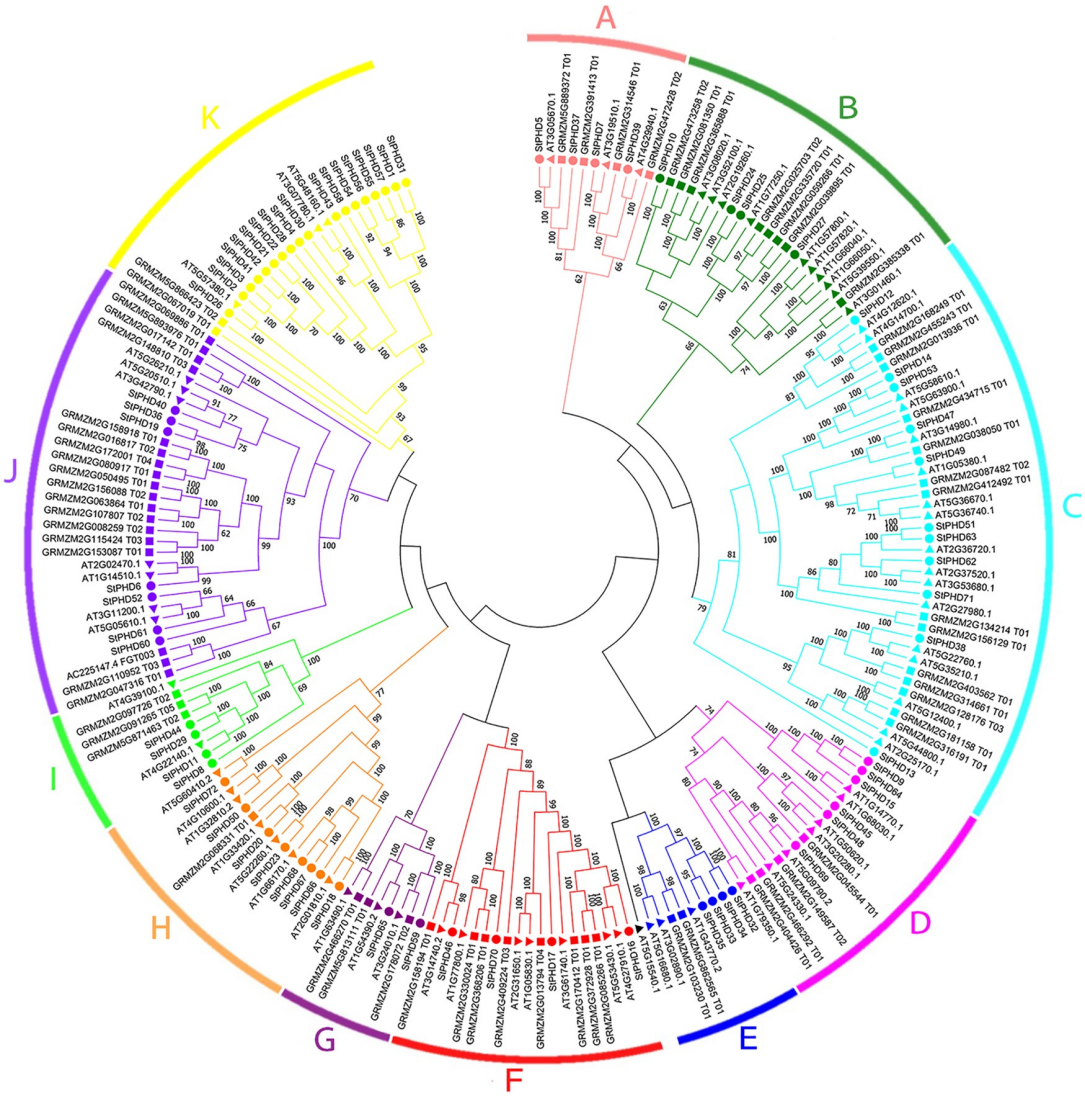
Phylogenetic analysis of potato, Arabidopsis and maize PHDs. The tree was constructed by maximum-likelihood method. Each PHD subfamily was marked by a specific color, except for black, which represented genes that did not belong to any subfamily due to low bootstrap values. The square, circle and diamond represent maize, potato and Arabidopsis PHD proteins, respectively.

To explore the evolutionary process of the *PHD* gene family in potato, 72 StPHD protein sequences were aligned with 70 PHD proteins from Arabidopsis [36] and 67 PHD proteins from maize [17]. The phylogenetic tree was classified into 11 distinct groups (group A through K) with high bootstrap values (≥60%) support (Fig 4). The PHD proteins with low bootstrap values were not included with any subfamily. Group C was the largest subfamily, with 42 proteins, whereas the smallest groups, E, G and I, contained 8 to 9 proteins. StPHD proteins were more closely related to those in the same subfamily from Arabidopsis and maize than those of the other potato PHD proteins. Recent reports showed that PHD proteins for many plant species were highly similar to those of Arabidopsis [43]. Based on our analysis, most PHD protein structures in potato, Arabidopsis and maize were highly similar. A total of 18 members of PHD subfamily K were identified and 5 times more members were found in potato than Arabidopsis; only 3 members were identified in Arabidopsis. Only 2 members of subfamily K were identified in maize and 8 more times were found in potato than in maize (Fig 4).

In total, 77 ortholog pairs (St-At) were identified between potato and Arabidopsis (S3 Table). However, only 68 ortholog pairs were found between potato and maize (St-Zm) (S4 Table). The number of orthologs between potato and Arabidopsis were greater than that between potato and maize, which may be due to the closer evolutionary distance between potato and Arabidopsis. One *StPHD* gene identified two or more orthologs from Arabidopsis or maize. For example, *AT1G14510*, *AT2G02470*, *AT3G42790*, *AT5G26210* and *AT3G11200* are orthologs to *StPHD6*, and these paralogs may perform important roles in the expanding process of the *PHD* gene family in plants.

### GO annotation for StPHD proteins

**Fig 5:**
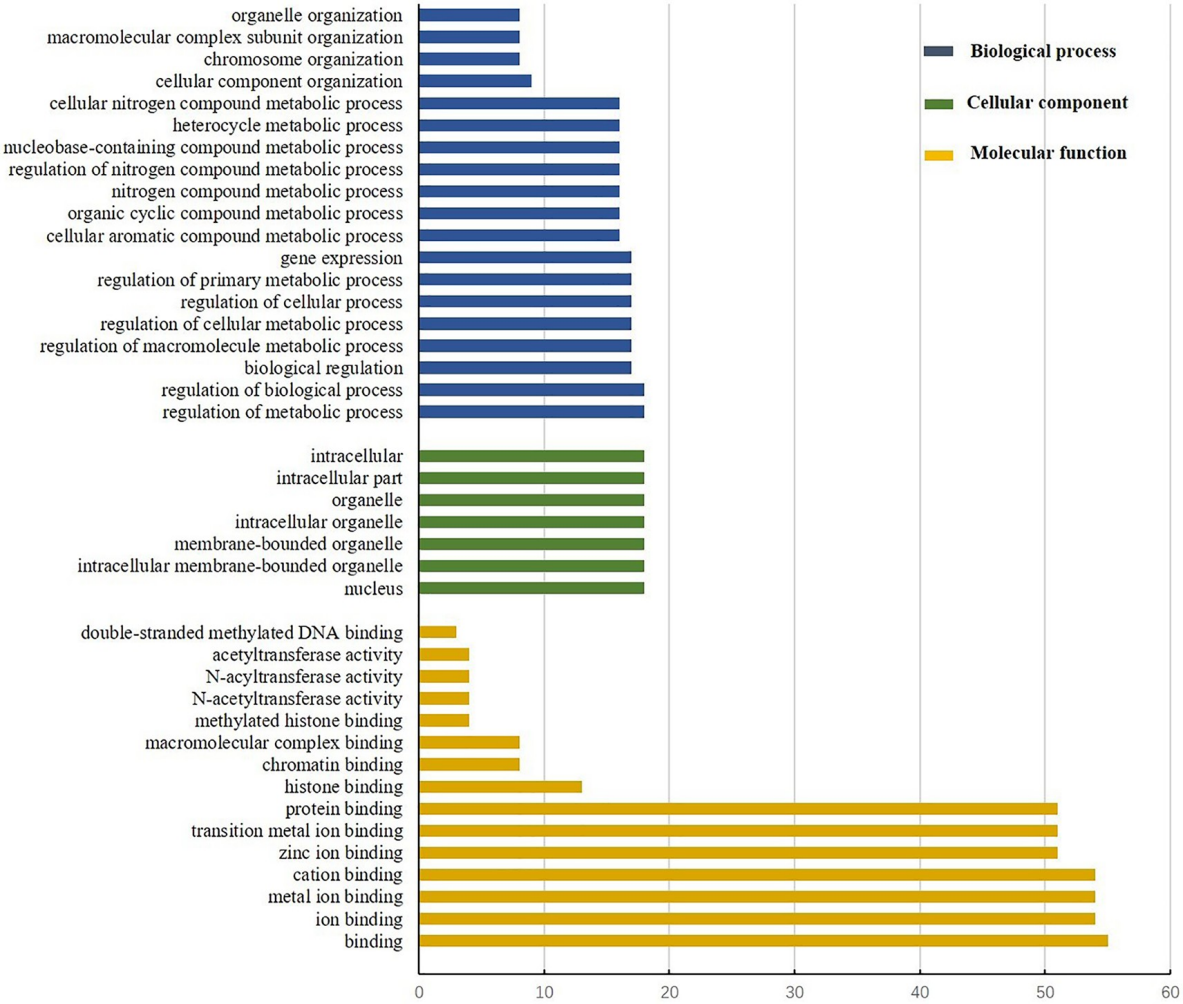
GO annotation of StPHD proteins. The annotation was performed on three categories, including biological process (blue color), cellular component (green color), and molecular function (yellow color).

Previous reports showed that PHD is involved in many biological processes at the nuclear level [4], including chromatin modification and mediation of molecular interactions in gene transcription. In addition, PHD displayed the complicated histone sequence reading ability mediated by histone modifications [44]. Diverse functions were found by the GO analysis of StPHD proteins (Fig 5). The majority of StPHD proteins were involved in intracellular activities, whereas some of them were located in the nucleus, others were located in the organelle. Some StPHD proteins had molecular function of binding, including ion, protein, DNA and chromatin. Meanwhile, some StPHD proteins showed function for acetyltransferase activity. In terms of biological processes, StPHD proteins participated in various biological pathways and regulated the various metabolic and biological processes, such as mediation of various organizations of cellular components, chromosomes, macromolecules, complex subunits, and organelles. In addition, some StPHD proteins engaged in gene expression. The diverse biological functions of StPHD proteins may be due to diverged sequences rather than the conserved StPHD domain.

### Tissue expression patterns for *StPHD* genes

**Fig 6:**
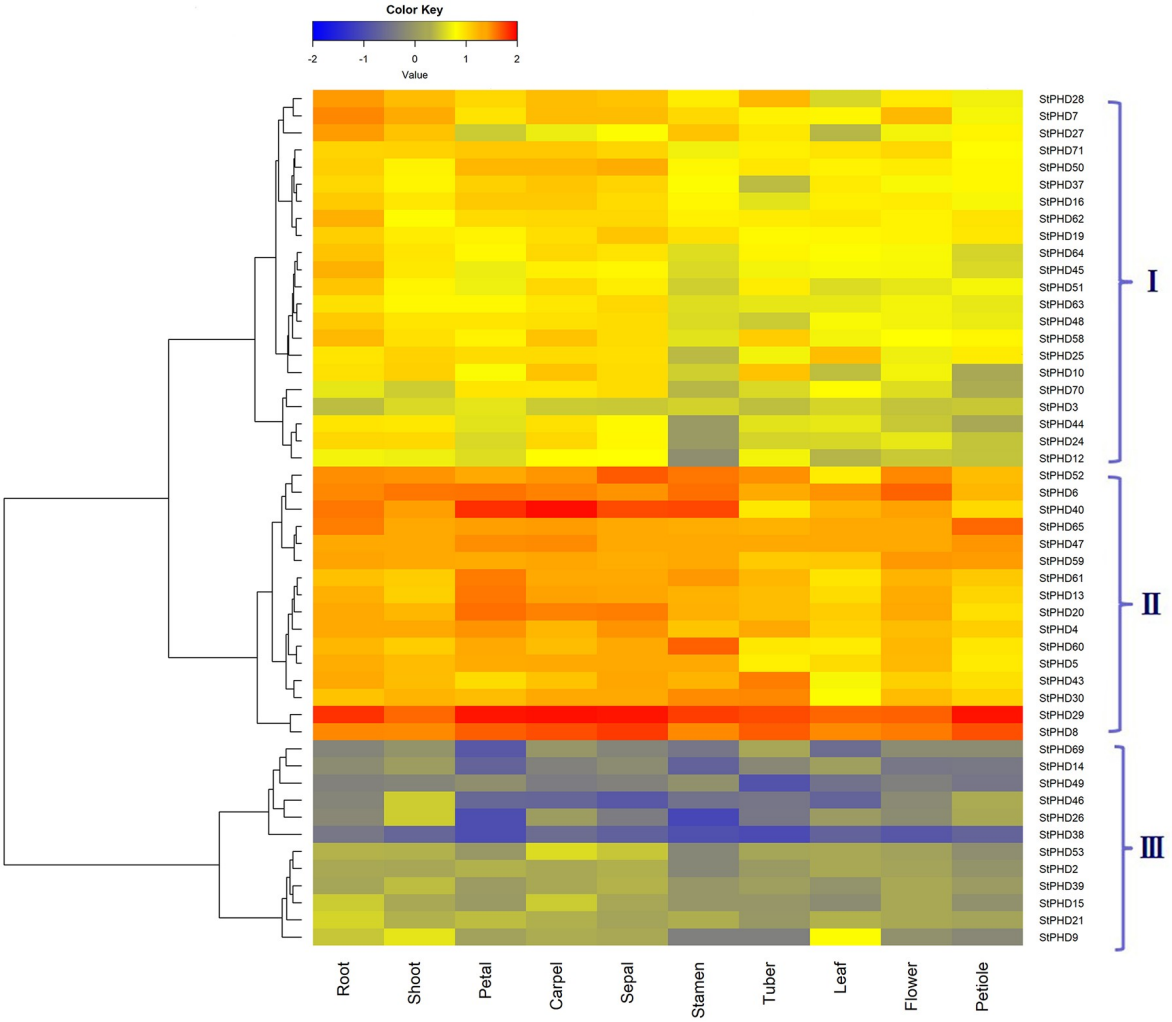
Expression heatmap of *StPHD* genes in different tissues. FPKM values for *StPHD* genes were transformed by log10.

To reveal the potential functions of the *StPHD* genes, the expression patterns of *StPHD* genes were investigated based on the FPKM values extracted from SpudDB from 10 main tissues (root, shoot, petal, carpel, sepal, stamen, tuber, leaf, flower and petiole) (Fig 6). However, 20 genes were excluded from the heat map analysis because of low expression (FPKM < 0.5) or lack of expression in all examined tissues. Based on the heatmap, considerable variations in expression were found among individual genes from different tissues. A total of 52 *StPHD* genes were clustered into 3 groups (Fig 6), and 16 genes (*StPHD*52, *-6*, *-40*, *-6*5, *-47*, *-59*, *-61*, *-13*, *-20*, *-4*, *-60*, *-5*, *-43*, *-30*, *-29* and *-8*) were included in group II, which showed high expression levels in most of the analyzed tissues. Among them, two genes (*StPHD29* and *StPHD8*) were especially abundant. In addition, 12 genes were included in group III and showed lower expression levels in most of the tissues. Some *StPHD* genes showed tissue-specific expression patterns. For example, *StPHD27* was found to show abundant expression in root, shoot and stamen but had lower expression levels in petal, carpel and leaf. The expression patterns for these genes provided preliminary information for their functions.

### Expression profiles for *StPHD* genes in response to abiotic stress

**Fig 7:**
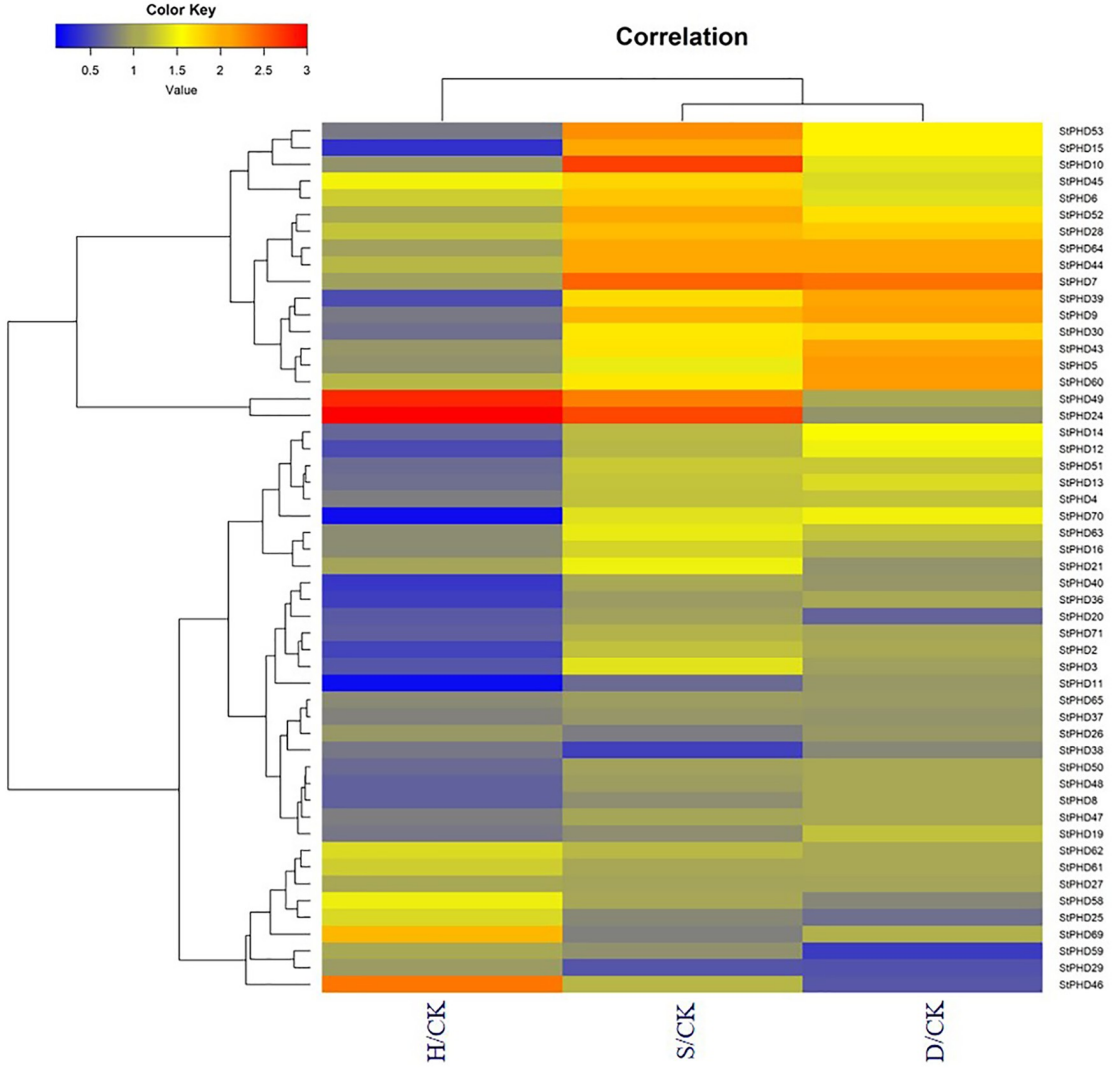
Expression heatmap for *StPHD* genes exposed to heat (H), salt (S) and drought (D) stress.

To further explore the response of the 52 *StPHD* genes following exposure to various types of stress (heat, salt and drought), a heatmap was generated using the relative expression level (represented by the FPKM values of stress/control) (Fig 7). In response to heat stress, most of the *StPHD* genes were downregulated. *StPHD15*, *StPHD39*, *StPHD70* and *StPHD11* showed significant downregulation, and a few *StPHD* genes (e.g., *StPHD49* and *StPHD24*) exhibited significant upregulation. In contrast, the variation in expression levels in response to salt and drought stress were not as divergent as that of heat stress. Most *StPHD* genes were upregulated; *StPHD10*, *StPHD7* and *StPHD24* displayed the most significant upregulation in response to salt. However, *StPHD59* showed significant downregulation in response to drought stress. A few *StPHD* genes (e.g., *StPHD10*, *StPHD69* and *StPHD46*), showed contrasting expression patterns among the three stress types. *StPDH46* was not sensitive to salt stress, but it was upregulated when exposed to heat stress and downregulated with drought stress. The *StPHD* genes with diverse stress responses and expression patterns may be due to the functional dissimilation of StPHD proteins.

**Fig 8:**
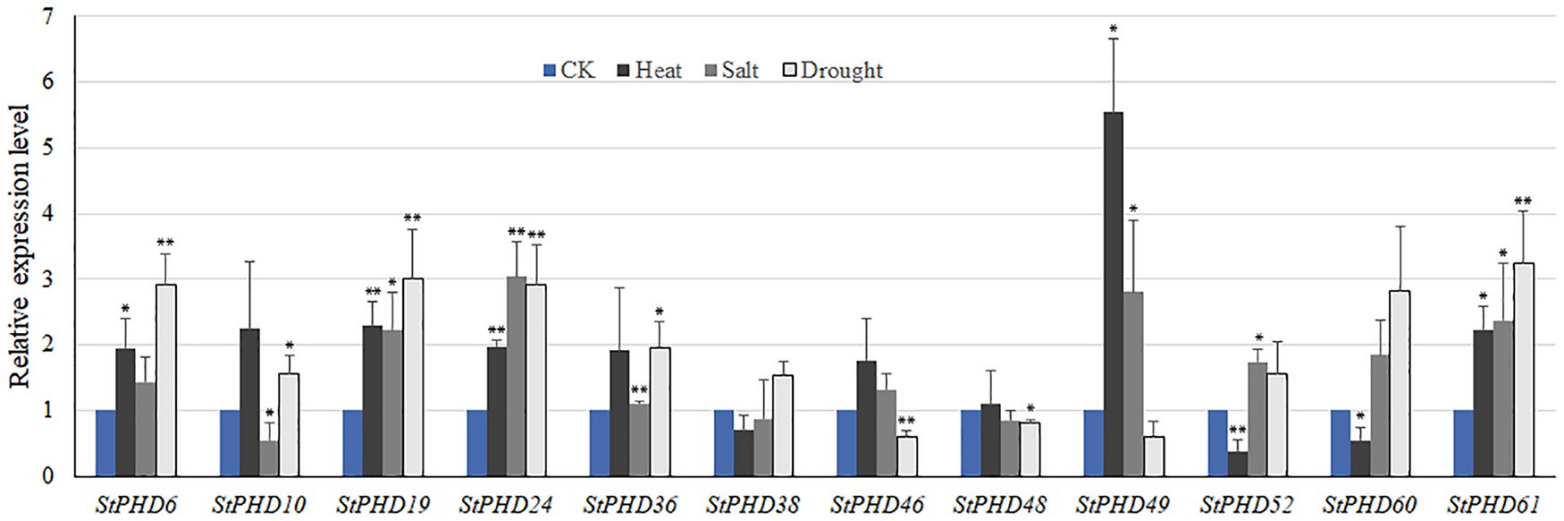
Relative expression level of 12 *StPHD* genes in response to heat, salt and drought stress at 6 h. The error bars indicated standard deviation. Asterisks indicated that the expression level was significantly different from the value of the control (*P<0.05, **P<0.01).

It has been confirmed that a subfamily of the maize *PHD* gene family was involved in abiotic stress response (namely, GRMZM5G893976, GRMZM2G017142, GRMZM2G148810, GRMZM2G158918, GRMZM2G016817, GRMZM2G172001, GRMZM2G080917, GRMZM2G050495, GRMZM2G156088, GRMZM2G063864, GRMZM2G107807, GRMZM2G008259, GRMZM2G115424, GRMZM2G153087, AC225147.4, GRMZM2G110952 and GRMZM2G047316) [17]. In our study, phylogenetic analysis showed that all of these members in this maize subfamily were distributed in group J (Fig 4), suggesting the members in this group shared a close evolutionary relationship. As the functions were related to their protein structures, we concluded the 7 *StPHD* genes (*StPHD40*, *-36*, *-19*, *-6*, *-52*, *-61* and *-60*) in group J (Fig 4) may also perform a similar role in response to various stress types in potato. Therefore, to further understand the function of these *StPHD* genes, qRT-PCR was used to study the expression patterns of 7 *StPHD* genes in response to abiotic stress (heat, salt and drought). In addition, 5 *StPHD* genes (*StPHD10*, *-24*, *-38*, *-46* and *-49*) that exhibited significant response to abiotic stress, based on the FPKM values from SpudDB (Fig 7), were also selected for qRT-PCR analysis. The expression patterns for the 12 *StPHD* genes in response to heat, salt and drought are shown in Fig 8. In total, *StPHD48* showed a slight response to all three abiotic treatments. Of the 12 PHD-finger genes, most of the genes were upregulated in response to heat stress, except *StPHD52* and *StPHD60*. *StPHD10*, *StPHD19 and StPHD61* were significantly upregulated (>2 fold). *StPHD49* showed extreme sensitivity (>5-fold) in response to heat stress (Fig 7). Although 9 genes were upregulated in response to salt treatment, *StPHD10*, *StPHD38* and *StPHD48* were slightly downregulated compared to that of the control. In response to drought stress, 5 (*StPHD6*, *StPHD19*, *StPHD24*, *StPHD60* and *StPHD61)* out of the 9 upregulated genes showed significant variation in expression level (>2 fold), whereas *StPHD46*, *StPHD48* and *StPHD49* were slightly downregulated.

## Discussion

Previously reported PHD proteins, which participate in many biological processes including chromatin modification and mediating the molecular interactions in gene transcription and histone modifications, played important roles for the growth and development of plants [4, 44]. To comprehensively analyze the *PHD* gene family, genome-wide identification and annotation of *PHD* genes have been performed for many species including moso bamboo (60), maize (67) [17], *Populus trichocarpa* (73) [18], rice (58) and *Arabidopsis* (70) [45]. The total members of PHD and genome size appeared not to be always related when the PHD gene family of the reported species was compared. For example, moso bamboo has much larger genome sizes than that of poplar and Arabidopsis but possesses fewer *PHD* genes than these two species. Therefore, *PHD* gene families may undergo differential expansion in different species. It was hypothesized that *PHD* gene family expansion might be related to three whole-genome duplication events in Arabidopsis [46] and the specific “Salicoid” duplication event in poplar [47]. In maize, the segmental duplication events were found to perform an important role in the amplification of the *PHD* gene family during the evolutionary process [17]. It was reported that potato genome had gone through at least two rounds of genome duplication; one ancient event might have occurred after the divergence between dicots and monocots approximately 185 ± 55 million years ago, and the recent duplication occurred approximately 67 million years ago [48]. In our study, a total of 72 nonredundant potato *PHD* genes were identified. Of these genes, 18.06% were identified from segmental duplication suggesting that whole genome duplication events performed essential roles during the amplification process of *PHD* gene family. Compared to random duplicated genes in maize (3.0%) and poplar (5.5%), more than two times the tandem duplicated genes (12.5%) were found in potato. These results suggest that the random duplicated event performed an important role during the *PHD* gene family expansion process in potato, which differed from that of maize and poplar. According to the phylogenetic tree (Fig 4), we found potato had many more members than Arabidopsis (5 times) and maize (8 times) in group K, furthermore, two paralog pairs (*StPHD2/3* and *StPHD21/22*) were derived from random duplicated events distributed in this subfamily. These results further support the important role of random duplicated events during the *PHD* gene family expansion process in potato.

Protein structure is closely linked with protein function. In our study, most of the StPHD proteins included in the same subfamily exhibited similar domain architectures, which indicated that they could perform similar functions. Furthermore, the structures and motif constitutions for the StPHD protein within each group were basically matched with the phylogenetic analysis (Figs 1 and 2). However, a few branches of the phylogenetic tree failed to categorize into any subfamily because of their low bootstrap values, which may be due to the relatively few feature positions except the highly conserved PHD domain. A similar phenomenon was found in other species, such as maize [17], Arabidopsis and poplar [18]. *StPHD* members appeared to have different patterns for gaining or losing introns since their origin, and the proteins in the same group were not entirely identical in terms of their structure and motifs (Figs 1 and 2). These results imply that the highly diverse characteristics of sequences among these genes, other than that of the conserved PHD-finger domain, have contributed to the functional diversity of the PHD in potato.

Based on the RNA-seq data for potato, the expression of *StPHD* genes exhibited incongruous expression profiles in various tissues (Fig 6). Two genes, *StPHD8* and *StPHD29*, showed the specific action of housekeeping expression and they displayed significant expression levels in most of the tissues. Studies for 6 pairs of tandem duplicated genes found that many of these paralogs (*StPHD22*, *-67*, *-68*, *-33*, *-34* and *-35*) exhibited lower or no expression level in the investigated tissues; these results suggest that they may be expressed at specific developmental stages or have gone through pseudogenization. Most paralogs displayed similar expression patterns, and only a few segmental duplicated *StPHD* gene pairs displayed diverse expression patterns with each other (e.g., *StPHD8* and *StPHD44*), suggesting that the functional differentiation may be a main character for the surviving duplicated genes [49]. It was reported that some PHD proteins were involved in abiotic stress responses [16]. For example, a subfamily of the maize *PHD* gene family was reported to be involved in abiotic stress response by RT-PCR [17]. According to the phylogenetic analysis, 7 StPHD proteins were grouped in the same cluster with these maize subfamily members (Fig 4), indicating that these 7 *StPHD* genes have the potential function of abiotic stress response in potato. This conclusion was verified by qRT-PCR results (Fig 8), and 7 *StPHD* genes showed varying response to heat, salt and drought treatments.

To analyze the trend of the gene expression derived from qRT-PCR (Fig 8) and the FPKM values, we compared the results from these two different platforms (Fig 7). Similar tendency of the gene expression was found between the two different platforms. Two genes (*StPHD6* and *StPHD10*) had the same expression trends in response to the three abiotic treatments. Four genes (*StPHD24*, *-38*, *-46* and *-49)* had similar expression patterns in response to heat and salt stress. *StPHD49* was extremely sensitive in both sets of results, whereas three genes (*StPHD52*, *-60* and *-61)* had similar expression patterns in response to salt and drought stress. For other genes, the same direction changes were identified in response to one of the abiotic stress types. However, the results of qRT-PCR did not totally agree with the pattern of gene expression from RNA-seq data, and this observation may be due to several putative reasons. First, the potato varieties used in the two experiments were different; a doubled monoploid potato variety (DM) was used in RNA-seq [21], whereas autotetraploid cultivar Zhongshu 3 was employed in qRT-PCR. Second, the experimental treatments were different. The plants for qRT-PCR were exposed to a photoperiod of 16 h light/8 h dark in this study, whereas the plants for RNA-seq were placed in the dark environment [21]. Third, the relative expression data was generated by using the 2^−ΔΔCt^ method depending on the SYBR, and the RNA-seq data used in the present study was indicated by FPKM. The FPKM value should be a good method to decrease sample differences, but it may cause deviation for highly expressed genes [50]. The bias of the FPKM value may result in different expression compared to that of qRT-PCR.

## Supporting information

S1 Table Potato *PHD* gene-specific primers used for qRT-PCR analysis. (DOCX)

S2 Table Sequences of 20 predicted motifs of StPHD proteins. (DOCX)

S3 Table Information about orthologs between potato and Arabidopsis. (XLSX)

S4 Table Information about orthologs between potato and maize. (XLSX)

S1 Fig Multiple sequence alignment of PHD-finger domain of 72 potato PHD proteins. Identical residues are labeled in black while similar residues are colored in pink, and the high conserved amino acids are positioned at the bottom of the figure.
(TIF)
