# Supplementary material for: Genome-wide identification and expression analysis of the PHD-finger gene family in Solanum tuberosum
Source: PLoS One. 2019 Dec 27;14(12):e0226964. doi: 10.1371/journal.pone.0226964 (PMC6934267; doi:10.1371/journal.pone.0226964)
Supplement: S1 Table — (DOCX) [file pone.0226964.s001.docx]

| **S1 Table** Potato *PHD* gene-specific primers used for qRT-PCR analysis. | | |
| --- | --- | --- |
| Primer Name | Forward Primer (5′→3′) Sequence | Reverse Primer (5′→3′) Sequence |
| StPH6 | TCATGGCTGCTTTCTGTTGC | GCAGCTCCGGTAACAACTTC |
| StPH10 | CCAGTGACAGAGTTTCGTGC | CAGCAGGCACACTCAAAAGA |
| StPH19 | GCAAAAGGACAGAAGAAGCCT | ACCTCCCATTGCTCATTTGG |
| StPH24 | CCCCTCTAGCTCAGTGATAATTG | ATTCGCCCTCAACTCCTTCA |
| StPH36 | ACTTGTGCTTGTATGGTCTCC | GGACAGCAACCAAGGATAGC |
| StPH38 | CCCTGATGGTGAACTGTCTG | ACCCCATGTAAAAGCAACCAC |
| StPH46 | TGTGGATGACGATGATGGGA | AGCACAAAACCAATCGCCTT |
| StPH48 | TGTCCAAGGTGCTTCAGGAT | AGCTAGAAGATGTACTGGGCT |
| StPH49 | GATGTCGTCAAAAGTCTGGTCT | AAAGCCCCTGAAATTCACCC |
| StPH52 | TCTTGCTTTTAGTTGCCGTCA | TGGTCAATCAAGCAAAAGCCA |
| StPH60 | TGGAGTGGATTTGGGTTTGT | CGAAACAAAACACAGAGGCG |
| StPH61 | CGTTGTTGTTCCTGAGTTGGT | GAGCAGTTACTTCGTAGCCG |
| StActin | GCTTCCCGATGGTCAAGTCA | GGATTCCAGCTGCTTCCATTC |
|  |  |  |
