# Supplementary material for: Genome-wide identification and expression analysis of the PHD-finger gene family in Solanum tuberosum
Source: PLoS One. 2019 Dec 27;14(12):e0226964. doi: 10.1371/journal.pone.0226964 (PMC6934267; doi:10.1371/journal.pone.0226964)
Supplement: S2 Table — (DOCX) [file pone.0226964.s002.docx]

**S2 Table** Sequences of 20 predicted motifs of StPHD proteins

| **Motif** | | **Width** | **Motif Sequence** | **Annotation** |
| --- | --- | --- | --- | --- |
| 1 | 75 | | VGGSGNLEMQYLCRGCDHRSELFGFVKDVFNSCAKVWSEETJEKELBYVRKIFRGSZDFKGKELHHKIESJKAKL | PHD_SF super family |
| 2 | 112 | | ELVSLQNRLNERSDLTNZTLLKCHKTQLEILVAIKTGLQSFLSENTHLSTSELIEIFLLERCRNINCRRVLPVEDCKCKICSTKKGFCSECMCLVCLKFDCANNTCSWVGCD | PHD_SF super family |
| 3 | 50 | | CKNYADDEFLILCDGCERWYHTRCVGITPAEAEEWKCYCCPCCSSKESRI | PHD_SF super family |
| 4 | 104 | | RTVEEVFKDYSARRAGJIKALTTDVEEFYNQCDPEKENLCLYGHPNETWEVNLPAEEVPPELPEPALGINFARDGMNEKDWLSLVAVHSDSWLLSVAFYFGARF | Alfin |
| 5 | 121 | | RPEHPPMQKEKDAIHDPREKKNCEVCGDFGIQEAIITCYQCKNVDVHQYCVVGYWEDAPVDWRCEECDIRKGVMFSPRGLENERFKGPKLHASTKICQSTVQPKKHSKFPRRQHINWEKEV | PHD_SF super family |
| 6 | 97 | | MNYGSNFNRLBFGGFYTVILEVNDEVVSAATVRIFGEEVAELPLVATRFEYRRQGMCRILMNSIEKLLMSLKVERLVLPAAPEAJETWTTSFGFRKI | NAT_SF |
| 7 | 90 | | NNVKVRVRWYYRPEESIGGRRQFHGAKELFLSDHYDVQSAHTIEGKCTVHSFKNYTKLENVGPEDYFCRFDYKAATGGFTPDRVAVYCKC | BAH super family |
| 8 | 67 | | PFPAMLCDICCSEPFFCRDCCCILCKKTTSSDYGGYYIRCEETTIDGYICGHVSHLDCALRAYMAGT | PHD_SF super family |
| 9 | 137 | | RSSRTSKDSTKSVSSSKLSRSSKRAVQVHSRTQLKHTKRDVKMHRJVFEEGGLPDGTEVAYYSRGKKLLEGYKKGAGIVCSCCNTEVSPSQFEAHAGSASRRPPYGYIYTSNGVSLHEFAIMLEKGRKSSTKDSDDL | Jas |
| 10 | 96 | | KTVEDEWSVKRSKKDZIDELESIVRIKEAEAQMFQSRADDARREAESLRRIARLKSEKLEEDYYEKLSKLRLQEAEERRRKKLEELKTLENSHSDY | Oberon_cc super family |
| 11 | 63 | | QAHPPCRVSRKVYEFSGLLPDTLKLELVPRGDIWPSLFDNHCPGKEDIGLYFFESEKKRFEGY | – |
| 12 | 54 | | RDGGGAYEFSRQQKILREIVSEPIPLMAQIVQZLPDETVESTKEYLRNLITMPE | – |
| 13 | 200 | | CAKAAKKFKQDVAGPIQNTVPDLISSQRDGVPAESCGGSQSIVQKRNSEDDGTLKANGCLKDGKAKHAASASVLCGIDSSIKDRLPQRMSSDKKFQNIESSSNCCAELGTSSDSSLRVIQQDSRTCGSKGDLDYNLPRDLQNGEPSNADKENIEQSRESEFSGDTDECHNESTDLASQKNDFLNLQYAQGEDSLATVGCT | – |
| 14 | 195 | | MSRQEARDAARLHIGDTGLIDYVLKSMNNVIIGGYVVRRAVNRATRVLEYTIQELRNCDQPEQEKLPEPFQDYAVNPGADAYTDVLCLYNNLLLSFAESDELSLAVRIVSDSKQFLKEWPFRDDPDDSLLRFICCILPSSNGLEAVFTKGYPPGEVVEVPLHSTIGDLKIAVESAMRGTYCIIDNLMITDIAGME | – |
| 15 | 70 | | RARVVYNGSASRINEIGLQLYPVSEYDSGEGLPYAPVDWPNAGDKWGWRVGKRVTSSGTFIDRYLYLPKH | – |
| 16 | 131 | | KNQQDKNTPDREKCWPLCALDLKGCDECCRAGRRKPEPNVAELVGDKVGFCDNNECKKQSKGRGWIFNRQGIKRRGWIEQQLDKGVKVEILCQHQPDNLNCMVLDWRNKADCQKLDCZSEYLFGWREKNKF | – |
| 17 | 79 | | LSGSDIMDFWDRLCTVFRARKISLEDVSKKYGMDLRLLHGVAYGHPWFGKWGYKFGRGSFGITZESYEKAIEALSSLPL | – |
| 18 | 98 | | LPWIWVIEALATSSEIDTSLLINLVKRTPEISDDLGRNAREMVSLRVLESLFVQKNSDANSVASVPGDKVELDPSRDCEDVLRCILLEVSASNLKTAT | – |
| 19 | 52 | | NKAERKRLFSMINDLPTIFEVVTGRKKIKQKPKADSGSKSKGSSKKRSSKGA | Alfin |
| 20 | 105 | | FGDLQPNFIEGAKRKGNFERDYDDHHSQVFQDGSESKMEQWKCZRCPIDGNDNDDCDGGIPGVHMLNHYDKDPRAFAQTSQPKPERRGEPGDCCEEDCNDDDPER | – |

–Means no annotation was found
