# Supplementary figures and images for: Genome-wide identification and expression analysis of the PHD-finger gene family in Solanum tuberosum
Source: PLoS One. 2019 Dec 27;14(12):e0226964. doi: 10.1371/journal.pone.0226964 (PMC6934267; doi:10.1371/journal.pone.0226964)

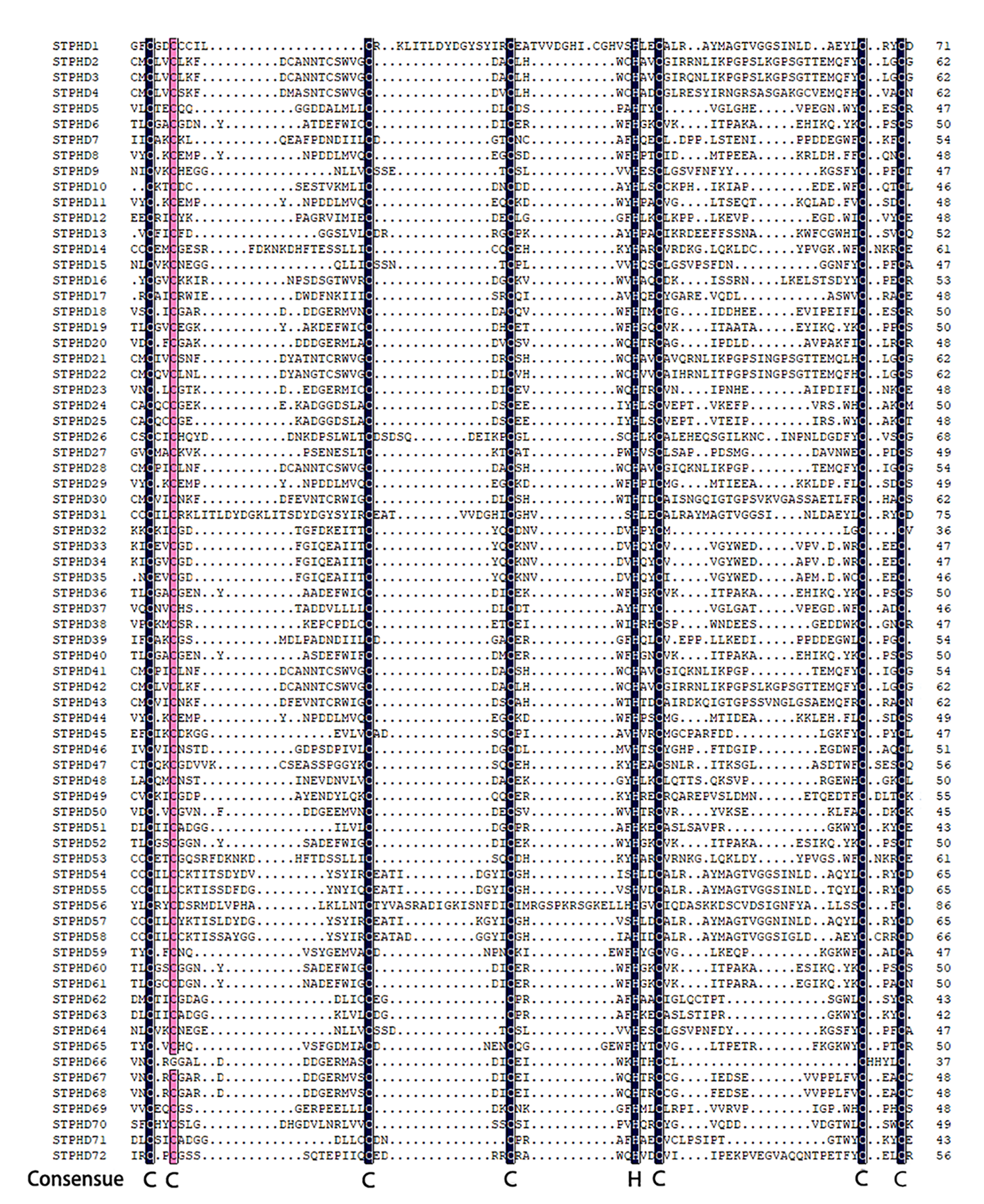

Supplement: S1 Fig — Identical residues are labeled in black while similar residues are colored in pink, and the high conserved amino acids are positioned at the bottom of the figure. (TIF) [file pone.0226964.s005.tif]
